# Key features and updates for Origin 2018

**DOI:** 10.1186/s13321-018-0259-x

**Published:** 2018-02-09

**Authors:** James G. Moberly, Matthew T. Bernards, Kristopher V. Waynant

**Affiliations:** 1 Department of Chemical and Materials Engineering, University of Idaho, 875 Perimeter Dr. MS 1021, Moscow, ID 83844-1021 USA; 2 Department of Chemistry, University of Idaho, 875 Perimeter Dr MS 2343, Moscow, ID 83844-2343 USA

## Abstract

OriginLab’s newest version update to Origin and OriginPro includes ease-of-use features, like Origin Central updates and creation of an App Center, as well as larger changes like the addition of Unicode characters, alteration to how user files are stored and visually searched, and user input formula in cells within worksheets. These features add additional value to an already powerful data analysis and plotting software package.

OriginLab’s latest update to their plotting and scientific software Origin and OriginPro 2018 (hereafter collectively referred to as “Origin”) provides some welcome updates for ease of processing data and usability as well as some new features which may change the way you use Origin.

OriginLab has made migrating between versions easier by moving the user files storage location outside of each Origin version’s root folder structure. Thus when upgrading to future (≥ 2018) versions (using default settings), all of your custom files should be automatically carried over without having to manually copy them over. Along with this upgrade Origin now uses .opju files (as well as the previous .opj filetypes for backward compatibility) which load more rapidly, are smaller in size, and enable some useful features within Microsoft Windows^®^ like representing project files in File Explorer with icon previews based on the active graphs, similar to viewing other photographs or images. This new feature should help users more easily sort and search their catalog of Origin project files, especially for automated batches.

One significant feature OriginLab added with the newest version is the addition of user input formulas in cells within worksheets, similar to other spreadsheet applications. Dragging alters the referenced cells unless specified as an absolute reference, just as in other spreadsheet applications. While still in the initial stages and not yet as full-featured as Microsoft Excel^®^, this may change how you utilize Origin and give it a more familiar feel for spreadsheet users. Not all of the functionality from Excel^®^ is present, and the structure and syntax is slightly different, but we believe this feature is a significant and important change to Origin and will make it even more useful as a graphing and data analysis package.

Origin now supports Unicode characters which enables users to select from a wide array of characters, and these can be used in worksheets, legends, and other fields. In our testing, the majority (but not all) of the four-character combinations worked and this may have been font library issues rather than Unicode/Origin issues. Simply enter the four-character code and press the Alt and X keys simultaneously to transform the sequence into a Unicode character. Font type Segoe UI Symbol and Lucida Sans Unicode seems to work well for nearly every Unicode character used. Now research is not hindered by different characters between user’s projects.

Origin is a powerful and full-featured data analysis software. Our research groups use a variety of the many features that Origin offers including linear and non-linear curve fitting, model validation, dataset comparison tools, and multi-dimensional data analysis. The powerful visual representation tools with accompanying descriptive statistical parameters help aid in hypothesis testing, model development and verification, and data analysis. Although coming from different backgrounds, Origin has helped our different research groups better collaborate and share research tools and user developed content with one another using a common software platform.

While statistical functions, batch data processing, and fitting functions are generally intuitive, the quality, diversity, and ease of plotting publication quality figures in Origin matches or surpasses other data analysis and graphing software packages. The addition of Origin Central, included since Origin 2017 and improved further in Origin 2018, provides some very nice features including sortable graph samples with example images, recently accessed or created projects, books, or graphs, and a learning center to provide both tutorials and videos on various aspects of Origin. The example images with graph samples is one of the most useful features as it allows you not only to see different ways of representing data, but also how to structure the data in Origin to generate the plot. Newer features in Origin include easily distributing graphs for balanced multi-panel figures as well as exporting a specific graphing region rather than splitting existing multi-panel figures into single panel graphs. Exporting graphs and plots to Microsoft Word^®^ was also added to Origin. Adding reference lines (or other user functions) based on user specified formulas are easier to insert within graphs. Additionally, enhanced legend customization including the ability to independently alter symbol size, an upgraded legend wizard, and Unicode characters improve this version of Origin.

An App Center was also introduced in Origin 2018 allowing you to search and easily update applications that provide ease of use functionality and features for Origin. These files, with the .opx filetype extension, are available within the Origin application and on the OriginLab website free of charge. OriginLab employees are willing and eager to partner with users to create custom applications that benefit the community of users. There are many different application functions, including adding SMILES molecular representations within spreadsheets and graph legends, simultaneous multiparameter and multi-model fitting with global parameters, importing map images, and more. Each one we tested was easy to navigate, intuitive, and updating an App is almost a one-click process.

System requirements for both 32-bit and 64-bit versions of Microsoft Windows^®^ 7, Windows^®^ 8, Windows^®^ 8.1, and Windows^®^ 10 include 2 GB of RAM, 2 GB of free hard disk space, a 2.4 GHz or higher Pentium-compatible processor, and Internet Explorer 9 or later to access Origin Central. Mac users can run Origin via Microsoft Windows^®^ and Boot Camp or through virtualization software.

OriginLab offers single-user node-locked, multi-user node-locked, and concurrent (floating) network licenses as well as a dongle license option. We have used Origin on Microsoft Windows^®^ 7, Windows^®^ 8.1, and Windows^®^ 10 with a concurrent network license, as well Microsoft Remote Desktop for Mac users to connect to a network virtualization of Windows^®^ 10 without issues. While we have not utilized the node-locked version with Origin 2018, we have used single-user node-locked with previous version of Origin (2016) and the activation and deactivation procedures for transferring Origin to a different computer are intuitive and painless. Origin also features a license borrowing option if you’ll be away from your network and can’t initiate a VPN, which is very useful for travel or field season. Borrowed licenses return to the pool of licenses when the computer reconnects to the network (if selected during checkout as an option).

For academic pricing (in USD), single user (node-locked) options start at $550 for Origin and $850 for OriginPro. A perpetual floating network license with three seats starts at $3450. Department (and multi-department), university, and classroom options are also available. Our multidisciplinary alliance benefited directly from a floating network license that allows both PI’s and students to collaborate seamlessly between research groups. Single user commercial licenses start at $1200 for Origin and $1900 for OriginPro. Government and other non-profit entities of Origin and OriginPro have licenses which cost $925 and $1525, respectively. Interested parties can securely purchase Origin directly through the OriginLab Store (store.originlab.com) or by contacting their local sales representative.

